# Resuming the discussion of AMSTAR: What can (should) be made better?

**DOI:** 10.1186/s12874-016-0183-6

**Published:** 2016-08-26

**Authors:** Uta Wegewitz, Beate Weikert, Alba Fishta, Anja Jacobs, Dawid Pieper

**Affiliations:** 1 Federal Institute for Occupational Safety and Health (BAuA), Nöldnerstr. 40-42, 10317 Berlin, Germany; 2 The Federal Joint Committee (G-BA), Wegelystr. 8, 10623 Berlin, Germany; 3 Institute for Research in Operative Medicine, Witten/Herdecke University, Ostmerheimer Str. 200 (Building 38), 51109 Cologne, Germany

**Keywords:** Systematic review, Methods, Decision making, Evidence-Based Medicine

## Abstract

**Background:**

Evidence syntheses, and in particular systematic reviews (SRs), have become one of the cornerstones of evidence-based health care. The Assessment of Multiple Systematic Reviews (AMSTAR) tool has become the most widely used tool for investigating the methodological quality of SRs and is currently undergoing revision. The objective of this paper is to present insights, challenges and potential solutions from the point of view of a group of assessors, while referring to earlier methodological discussions and debates with respect to AMSTAR.

**Discussion:**

One major drawback of AMSTAR is that it relies heavily on reporting quality rather than on methodological quality. This can be found in several items. Furthermore, it should be acknowledged that there are now new methods and procedures that did not exist when AMSTAR was developed. For example, the note to item 1 should now refer to the International Prospective Register of Ongoing Systematic Reviews (PROSPERO). Furthermore, item 3 should consider the definition of hand-searching, as the process of reviewing conference proceedings using the search function (e.g. in Microsoft Word or in a PDF file) does not meet the definition set out by the Cochrane Collaboration. Moreover, methods for assessing the quality of the body of evidence have evolved since AMSTAR was developed and should be incorporated into a revised AMSTAR tool.

**Summary:**

Potential solutions are presented for each AMSTAR item with the aim of allowing a more thorough assessment of SRs. As the AMSTAR tool is currently undergoing further development, our paper hopes to add to preceding discussions and papers regarding this tool and stimulate further discussion.

## Background

Evidence syntheses, and in particular systematic reviews (SRs), have become one of the cornerstones of evidence-based health care. If SRs are methodologically sound, they are considered to provide the highest level of evidence for medical decision-making. Over recent years, the Assessment of Multiple Systematic Reviews (AMSTAR) tool has become the most widely used tool for investigating the methodological quality of SRs [1, 2]. It was developed based on the Overview Quality Assessment Questionnaire [3, 4] and the checklist by Sacks [5] and consists of 11 items, each of which is categorized into a standardized set of four possible responses: “yes”, “no”, “can’t answer” or “not applicable”. The items relate to a priori design, study selection and data extraction, the literature search, grey literature, the list of included and excluded studies, study characteristics, critical appraisal, the formulation of conclusions, the combination of study results, publication bias and conflicts of interest. The measurement properties were recently described in a SR and found to be satisfactory in terms of interrater reliability, validity and applicability (scoring takes between 10 and 20 minutes), although it has also been stated that some methodological issues are still open to discussion, such as the test–retest reliability and whether and how an overall score should be calculated [6]. It should also be noted that several authors have modified or augmented the original AMSTAR tool, for example by adding new items, splitting items, or altering the rationales for answering or scoring items [7–11].

Two recently published papers present AMSTAR from the perspective of an assessor [12, 13]. Both papers describe challenges that the assessors faced when applying AMSTAR to SRs and present potential solutions to these challenges.

The main idea of our paper is to continue the discussion raised by these papers and to present another perspective from a different group of assessors. In doing so, we will focus only on the evaluation of SRs of randomized controlled trials (RCTs) using AMSTAR.

This is an ideal time for discussion, as the AMSTAR tool is currently undergoing further development. According to the developers of AMSTAR, this development will be two-fold [14]. Firstly, a new version of AMSTAR will improve upon the original tool. Secondly, a version of AMSTAR will be developed for SRs of non-randomized studies; this will be called AMSTAR-NRS. According to the tool’s developers, AMSTAR can be applied to a wide variety of SRs, although they recognize that its original development only took account of SRs of RCTs that evaluated treatment interventions [15].

The further development of AMSTAR is a sensible step. Even in the first AMSTAR paper, the tool’s developers acknowledged that new evidence would modify current thinking and that an update would be inevitable [16]. Now that AMSTAR is to be updated and further developed, it would make sense to gather the experiences of AMSTAR users from around the world, as these might provide important ideas that could be taken into account in the new, updated version of the tool.

The following discussion is structured according the order of the AMSTAR items, and each item is discussed separately. A comparison with other comparable AMSTAR discussion papers is included at the end of the discussion.

## Discussion

1. *Was an ‘a priori’ design provided? The research question and inclusion criteria should be established before the conduct of the review. Note: Need to refer to a protocol, ethics approval, or pre-determined/a priori published research objectives to score a “yes.”* [17].

An a priori protocol is an important component of SRs. The intention is to reduce the risk of bias related to selective reporting by stating a priori hypotheses and methods explicitly [18]. However, a thorough evaluation should focus on discrepancies between the protocol and the final review, which research has shown to be fairly common [19, 20]. It is not sufficient only to refer to a protocol, therefore, as this would primarily reflect the quality of reporting. In addition, clarification should be given of which aspects are included in a protocol or publication that is published a priori.

Times have changed since the development of AMSTAR. Some years ago, it was quite common for protocols typically only to be available for Cochrane reviews. In recent years, there have been many efforts to increase the quantity of systematic review protocols. It is worth mentioning the creation of the International Prospective Register of Ongoing Systematic Reviews (PROSPERO), which was launched in February 2011 [21]. This allows the documentation of 22 mandatory and 18 optional items with regard to the a priori design and conduct of a review. At the time of writing (February 2016), approximately 10,000 records (including Cochrane protocols) can be found in PROSPERO. It would probably make sense to mention PROSPERO as an example under this item. Furthermore, Preferred Reporting Items for Systematic Review and Meta-Analysis Protocols (PRISMA-P) were published last year [22], and SR protocols are published by journals such as Systematic Reviews, for example.

2. *Was there duplicate study selection and data extraction? There should be at least two independent data extractors and a consensus procedure for disagreements should be in place. Note: 2 people do study selection, 2 people do data extraction, consensus process or one person checks the other’s work.* [17].

This item addresses the two critical aspects of study selection and data extraction. Study selection should be performed by two people independently according to AMSTAR. It is important to bear in mind that there are also new approaches such as “liberal accelerated study selection,” in which the second reviewer reviews only the items that were excluded by the first reviewer [23]. Erroneously included records can still be excluded at a later stage. So far, this approach has been more prevalent in rapid reviews than in SRs. However, there is no obvious reason why this method should introduce bias into SRs.

AMSTAR provides for the possibility of one author checking the data extracted by another. This procedure is seen as error-prone because the quality of data extraction is not as good as if the reviewers had acted independently [24]. However, to the best of our knowledge, this is the only study that has investigated the issue of double data extraction; accordingly, the evidence is very limited. Furthermore, the reviewers’ levels of expertise might be presumed to influence data extraction. For instance, an inexperienced reviewer might check the work of an expert in a much more lenient manner than would be appropriate. This item will also need to be updated for future applications, as automated (computerized) data extraction is bound to emerge as an issue in systematic reviews [25]. In such situations, a human could check the correctness and completeness of the extracted data.

As a concluding remark, we would like to point out that the inclusion of two different aspects (study selection and data extraction) within one item might unnecessarily impede the application of AMSTAR in the event that only one of the two aspects is fulfilled.

3. *Was a comprehensive literature search performed? At least two electronic sources should be searched. The report must include years and databases used (e.g., Central, EMBASE, and MEDLINE). Key words and/or MESH terms must be stated and where feasible the search strategy should be provided. All searches should be supplemented by consulting current contents, reviews, textbooks, specialized registers, or experts in the particular field of study, and by reviewing the references in the studies found. Note: If at least 2 sources + one supplementary strategy used, select “yes” (Cochrane register/Central counts as 2 sources; a grey literature search counts as supplementary).* [17].

For the sake of reproducibility and to ensure comprehensive assessment of the literature search strategy, assessors should have access to details of the entire search strategy. However, AMSTAR simply requires the reporting of keywords and MESH terms for the awarding of a “yes” judgement. The full search strategy is to be set out “where feasible” but it is not obvious what this means. Today, it is possible in most cases to set out the full search strategy in an online supplement or at least to make it available upon request. As a matter of common practice, therefore, review authors provide the full search strategy.

Another problem centres around whether supplementary search strategies are adequate. In our opinion, the type of supplementary strategy used in SRs forms a critical aspect of a comprehensive literature search. In the case of SRs of clinical studies, for example, authors should focus their search on clinical study registers rather than on screening text books or dissertations. Furthermore, it should be clarified that it is mandatory for review authors to review the references in the identified studies. We therefore propose that the note be expanded as follows: *If at least 2 sources + one adequate supplementary strategy were used, and references of the included studies were screened, select “yes”*.

Furthermore, it should be borne in mind that different authors might have different definitions of hand-searching. Nowadays, conference proceedings can also be reviewed by using the search function, as most conference proceedings are available as PDF files; this falls under the definition of hand-searching given by the Cochrane Collaboration.

4. *Was the status of publication (i.e. grey literature) used as an inclusion criterion? The authors should state that they searched for reports regardless of their publication type. The authors should state whether or not they excluded any reports (from the systematic review), based on their publication status, language etc. Note: If review indicates that there was a search for “grey literature” or “unpublished literature,” indicate “yes.” SINGLE database, dissertations, conference proceedings, and trial registries are all considered grey for this purpose. If searching a source that contains both grey and non-grey, must specify that they were searching for grey/unpublished lit.* [17].

This item addresses multiple aspects of study selection: the publication status, the publication type, and language restrictions. Some concerns are associated with it. In order to fulfil this item, review authors need to search for grey literature. However, all that is required is a statement of whether or not the publication type and language restriction were used as inclusion criteria. Ignoring studies written in languages other than English could introduce a risk of bias. However, evidence shows that omitting non-English articles may have only a small effect, if any, on the conclusions of a systematic review [26, 27]. Nevertheless, it appears to be important to bear the research objective in mind; in other words, the preceding statement might not apply to research areas with a high prevalence of non-English articles and research [27, 28].

Furthermore, if review authors searched databases that appear to contain grey literature but did not mention this explicitly, AMSTAR recommends a “no” rating. This seems to be a questionable choice, as it is obvious that this item might in fact have been fulfilled. In conclusion, this item tends to evaluate the reporting quality rather than the methodological quality.

5. *Was a list of studies (included and excluded) provided? A list of included and excluded studies should be provided. Note: Acceptable if the excluded studies are referenced. If there is an electronic link to the list but the link is dead, select “no.”* [17].

This item covers the documentation of included and excluded studies. In our opinion, it would be better to split the item. Complete documentation of excluded studies is an important aspect that review authors seldom fulfil. This should therefore be addressed in a single, additional item, as most recently published SRs provide a full list of included studies. However, we are aware that this would yield a large number of “yes” responses for the documentation of included studies and therefore to little variation across ratings.

Another strategy for the documentation of excluded studies is proposed by the Cochrane Collaboration. According to the Cochrane Handbook, the list should not include all studies retrieved by the literature search but should contain studies that the reader might expect to be included [29]. In more specific terms, this means that Cochrane review authors present studies that are eligible at first glance but do not meet the eligibility criteria. In addition, well-known reports will also be considered if they are likely to be relevant to some readers. One can assume that this strategy introduces some subjectivity to the documentation process, which in most cases will not be reproducible by review assessors. We propose obligatory documentation of all studies excluded after the full text versions are evaluated. A description of other relevant studies may be helpful for the reader, and this could be presented on a voluntary basis.

6. *Were the characteristics of the included studies provided? In an aggregated form such as a table, data from the original studies should be provided on the participants, interventions and outcomes. The ranges of characteristics in all the studies analyzed e.g., age, race, sex, relevant socioeconomic data, disease status, duration, severity, or other diseases should be reported. Note: Acceptable if not in table format as long as they are described as above.* [17].

This item is one of the most subjective, as there is no defined threshold for assigning a “yes” or “no” rating. In our experience, the majority of reviews meet the minimum requirements for this item, resulting in a high proportion of “yes” answers.

7. *Was the scientific quality of the included studies assessed and documented? ‘A priori’ methods of assessment should be provided (e.g., for effectiveness studies if the author(s) chose to include only randomized, double-blind, placebo controlled studies, or allocation concealment as inclusion criteria); for other types of studies alternative items will be relevant. Note: Can include use of a quality scoring tool or checklist, e.g., Jadad scale, risk of bias, sensitivity analysis, etc., or a description of quality items, with some kind of result for EACH study (“low” or “high” is fine, as long as it is clear which studies scored “low” and which scored “high”; a summary score/range for all studies is not acceptable).* [17].

We believe that this item would benefit from some additional guidance. In our understanding, the item will most likely reflect reporting quality if the assessors simply look at whether an assessment has been made of the scientific quality of the included studies. The item should therefore ask whether the methodological quality has also been adequately assessed. The response will to some extent be subjective, as adequacy can be judged differently by different people. This might not be as important for SRs of randomized controlled trials, where the Cochrane Risk of Bias Tool can be used as a standard. In our opinion, the choice of the most adequate tool is not clear in the case of non-randomized studies. For example, the Newcastle–Ottawa Scale has been recommended by some journals but has also been criticized by others [30, 31].

It is important to add that the term “scientific quality” could be misleading in this context. This is because reporting quality might also be regarded as a form of scientific quality despite not being the focus of this item.

8. *Was the scientific quality of the included studies used appropriately in formulating conclusions? The results of the methodological rigor and scientific quality should be considered in the analysis and the conclusions of the review, and explicitly stated in formulating recommendations. Note: Might say something such as “the results should be interpreted with caution due to poor quality of included studies.” Cannot score “yes” for this question if scored “no” for question 7.* [17].

With regard to the integration of the methodological quality of individual studies, SR authors rarely provide a definition, or a good description, of the process of deriving conclusions from studies’ results. Different groups of authors might draw different conclusions. To improve the plausibility of this step, we propose not only assessing the quality of individual studies but also discussing the quality of the body of evidence. Quality of evidence describes how confident one can be that the estimate of an intervention’s effectiveness is true [32]. An evaluation of the body of evidence allows a link to be drawn between the quality of the overall evidence and the strength of the conclusions. Multiple tools exist for assessing and characterizing the quality of a body of evidence [33]. When the quality assessment of the body of evidence is integrated into the AMSTAR checklist, examples should be given of existing methods, e.g. the system proposed by the Agency for Healthcare Research and Quality (AHRQ) [34] or by the Grading of Recommendations Assessment, Development and Evaluation (GRADE) Working Group [35].

9. *Were the methods used to combine the findings of studies appropriate? For the pooled results, a test should be done to ensure the studies were combinable, to assess their homogeneity (i.e., Chi-squared test for homogeneity, I2). If heterogeneity exists a random effects model should be used and/or the clinical appropriateness of combining should be taken into consideration (i.e., is it sensible to combine?). Note: Indicate “yes” if they mention or describe heterogeneity, i.e., if they explain that they cannot pool because of heterogeneity/variability between intervention*s. [17].

This item appears to be useful and easy to understand but suffers from some ambiguities. For example, in the case of strong clinical or statistical heterogeneity, the problem of heterogeneity might not be solved by using the random effects model. Readers are often faced with SRs that highlight substantial heterogeneity between the included studies. But how should these SRs be evaluated when the authors have conducted a meta-analysis without discussing the appropriateness of combining the studies? AMSTAR does not provide guidance for such cases. The item could therefore be improved by requiring SR authors to state or discuss the criteria used for qualitative and quantitative synthesis of the body of evidence [36]. The choice of whether to conduct qualitative or quantitative synthesis is not always an obvious one. For example, two SRs examined the effectiveness of qigong for chronic conditions in the elderly [37, 38]. Given the similarity of the two SRs, one would expect both to have used the same synthesis method. In fact, one SR performed a qualitative synthesis, while the other performed a quantitative synthesis without giving any rationale for this choice. Furthermore, clinical expertise in the research topic is required in order to evaluate a SR and especially when judging the clinical heterogeneity of the included studies.

There may also be some obstacles to reaching a consensus on the assessment of reviews without meta-analyses. In this context, an explanation should be provided of whether this item is relevant to all SRs or only to SRs with a meta-analysis. Some authors provide a judgment of this item in all cases, while others state that it is not applicable in the case of narrative synthesis. Is it sufficient to cite clinical heterogeneity between studies without further explanation? In order to receive a score of “yes”, review authors should assess both the clinical and the statistical heterogeneity. Furthermore, we would like to point out that SRs can contain more than one pooled effect measure, i.e. multiple meta-analyses. This is often the case if more than one outcome is investigated and a meta-analysis is performed for each outcome. In such cases, it may be difficult to judge this item at the review level. Instead, it might be more appropriate to reach a judgment at the outcome level, which would make it easier to reflect differences in this item. For example, a SR might have applied fixed effect model meta-analysis appropriately to a number of outcomes. It would not be appropriate, however, to apply fixed effect model meta-analysis to all outcomes, as the best term to reflect the resulting judgment would probably be “partly”, which is not one of the possible answers under AMSTAR. We therefore believe that item 9 should ideally be answered at the outcome level. This would also be in line with the Cochrane Risk of Bias Tool or with GRADE [39].

10.  
*Was the likelihood of publication bias assessed? An assessment of publication bias should include a combination of graphical aids (e.g., funnel plot, other available tests) and/or statistical tests (e.g., Egger regression test, Hedges-Olken). Note: If no test values or funnel plot included, score “no”. Score “yes” if mentions that publication bias could not be assessed because there were fewer than 10 included studies.* [17].

In accordance with the above note, reviews with fewer than 10 included studies should receive a “yes” rating, provided that the authors stated that there were too few studies for an assessment of publication bias. In such cases, there is a high risk that reporting quality will be assessed instead of methodological quality. SRs that have fulfilled this item may vary substantially with respect to the assessment of publication bias. Though the difference between 9 and 10 may in fact be small, we appreciate that the AMSTAR tool provides a clearly defined threshold. Nonetheless, the idea that publication bias cannot be assessed due to a small number of studies means it is worth considering “not applicable” as a possible judgment.

Furthermore, this item should be given a score of “yes” in the case of SRs that assess the quality of evidence using GRADE methodology [40]. One possible justification of this judgment is that GRADE requires its users to assess the likelihood of publication bias. However, some review authors simply provide the GRADE Summary of Findings Table without addressing the issue of publication bias. In such cases, we would appreciate further guidance on how to judge this item. In our opinion, in order to fulfil this item, SR authors ought to discuss the efforts and methods they used to assess publication bias even if they applied the GRADE methodology.

In addition, we propose that assessors not only evaluate funnel plots or statistical tests but also consider the search strategy and/or potential conflicts of interest [41].

11.  
*Was the conflict of interest included? Potential sources of support should be clearly acknowledged in both the systematic review and the included studies. Note: To get a “yes,” must indicate source of funding or support for the systematic review AND for each of the included studies.* [17]

This item clearly addresses the potential conflicts of interest both in the review itself and in the included studies. However, it emphasizes the sources of funding or support, whereas readers need also to be aware of any personal conflicts of interest. One example of personal involvement is the inclusion of studies undertaken by a review author. To minimize the potential for influence in judgments, the assessment of eligibility and risk of bias in primary studies should be carried out by an author who was not involved [42].

Generally, this item is seldom fulfilled even in Cochrane reviews (which are known to be of high quality). This is not surprising, as neither PRISMA (Preferred Reporting Items for Systematic Reviews and Meta-Analyses) [43] nor the Cochrane Handbook requires the reporting of funding sources for trials.

We warmly welcome more-detailed assessment of conflicts of interest. This item should reflect the risk of bias due to conflicts of interest instead of simply examining whether they are reported, although we openly acknowledge that this task is a highly challenging one.

## Comparison with other assessors’ opinions

Recently, two other debate articles have been published regarding AMSTAR that provide a discussion at the item level [12, 13]. Both articles are based on the author’s experiences with AMSTAR. Faggion Jr. focused on two points in his analysis: the ability of AMSTAR to assess the methodological quality of SRs and challenges in the interpretation of items and their scoring guidance. He also provided suggestions for improving the tool [12]. Burda et al. followed the same idea, while also providing suggestions for rewording items and their scoring guidance [13]. As they did this for all 11 items, the results could also be referred to as a Burda version of AMSTAR. In addition, they also discuss the relevance of AMSTAR response categories.

For item 1, we placed greater emphasis on a published protocol rather than on an explicit statement in the review that review questions and inclusion criteria were defined a priori. There is still some debate as to whether checking someone else’s data extraction is an acceptable method of double data extraction given the lack of evidence in this field. With respect to item 2, all authors agree that it should be made much clearer that study selection and data extraction should be performed by two people. All three publications take the same approach to item 3. Faggion Jr. also presents a special emphasis on hand-searching [12], while only our manuscript strictly argues for the compulsory reporting of search strategy, assuming that this is feasible in all cases nowadays. Item 4 definitely needs to be reworded, as stated by all three publications, but while Faggion Jr. focuses on the number of sources [12], Burda et al. join us in highlighting the issue that this item is heavily reliant on reporting [13]. With respect to item 5, Faggion Jr. criticizes that this item does not state clearly whether the list of excluded studies should include hits from the stages of title/abstract screening or from the stage of full text evaluation [12]. He proposes reporting the full list of excluded studies from both stages, which is in line with the recommendation given by the PRISMA Statement [44]. However, a sensitive search strategy can result in a huge number of hits. From our point of view, we would like to question whether the benefit of this information justifies the huge effort involved in documenting the reasons for exclusion at the title/abstract screening stage. This concurs with Burda et al., who suggest only reporting the results of the full text screening [13]. Again, it is our opinion and the opinion of Burda et al. that item 6 will remain highly subjective [13], while Faggion Jr. proposes a threshold for a minimum amount of information [12]. In our view, this might not be feasible, as the minimum amount of information will probably depend on the objective of the review and the included studies. With respect to item 7, all of the publications recognise a need for revision. As a whole, it should be clarified that this item is about the risk of bias and that a “yes” score can only be obtained if an appropriate tool is selected for critical appraisal. With regard to item 8, Burda et al. also mention the GRADE approach [13], which Faggion Jr. had already mentioned in relation to item 7 [12]. Overall, all three publications arrive at a recommendation for the use of GRADE in SRs. There is a great deal of discussion with respect to item 9. While Faggion Jr. highlights the problems of quantitative synthesis [12], Burda et al. also make some points with regard to qualitative synthesis [13]. However, our manuscript is alone in calling for this item to be assessed at the outcome level rather than at the review level because heterogeneity can vary depending on the item being studied. The item concerning publication bias (item 10) raised discussions about the mixing methodological quality and reporting quality, as mentioned by Faggion Jr. [12] and by our group. This also raises the question of how this item should be rated. In this context, reference is made to GRADE both by Burda et al. and in our publication [13]. In general, all three author groups agree that item 11 must be clarified (e.g. that COI should be presented for all review authors), although there are differences regarding the degree of rigour needed to obtain a “yes.”

## Summary

This debate paper presents methodological reflections in relating to AMSTAR, a validated and widely used tool for evaluating the methodological quality of SRs. It provides insights and challenges from the point of view of a group of assessors, while referring to earlier methodological discussions and debates with respect to AMSTAR. Potential solutions are presented for each AMSTAR item with the aim of allowing a more thorough assessment of SRs.

As the AMSTAR tool is undergoing further development, our paper hopes to add to preceding discussions and papers regarding the AMSTAR tool and to stimulate further discussion.

## Abbreviations

AMSTAR, Assessment of Multiple Systematic Reviews; PROSPERO, International Prospective Register of Ongoing Systematic Reviews; PRISMA, Preferred Reporting Items for Systematic Reviews and Meta-Analyses; RCTs, Randomized controlled trials; SRs, systematic reviews
